# Prognostic role of body composition in peritoneal carcinomatosis patients undergoing cytoreduction and hyperthermic intraperitoneal chemotherapy

**DOI:** 10.1186/s12957-023-03233-0

**Published:** 2023-10-27

**Authors:** Young Song, Myung Il Bae, Dong Woo Han, Eun Jung Park, Sujung Park, Sung Yeon Ham

**Affiliations:** 1 Department of Anesthesiology and Pain Medicine, Yonsei University College of Medicine, 211 Eonjuro, Gangnam-Gu, 06273 Seoul, Republic of Korea; 2 Anesthesia and Pain Research Institute, Yonsei University College of Medicine, Seoul, Republic of Korea; 3 Division of Colon and Rectal Surgery, Department of Surgery, Yonsei University College of Medicine, Seoul, Republic of Korea

**Keywords:** Bioelectrical impedance analysis, Hyperthermic intraperitoneal chemotherapy, Prognosis

## Abstract

**Background:**

Bioelectric impedance analysis (BIA)-measured body composition and nutritional status have been used as prognostic indicators in various cancer cohorts. This study investigated whether BIA could provide information on prognosis in peritoneal carcinomatosis patients undergoing cytoreductive surgery (CRS) and hyperthermic intraperitoneal chemotherapy (HIPEC).

**Methods:**

We retrospectively analyzed the data of 99 patients with preoperative BIA data among those who underwent CRS and HIPEC. The association between BIA-derived parameters and intraoperative peritoneal cancer index (PCI) score was assessed. Predictive analysis for the occurrence of postoperative morbidities including major complications (Clavien–Dindo classification 3–4) and re-admission within 30 days after surgery as well as 1 year mortality was also performed.

**Results:**

BIA-derived mineral (*r* = 0.224, *p* = 0.027), fat (*r* =  − 0.202, *p* = 0.048), and total body water (TBW)/fat-free mass (FFM) (*r* =  − 0.280, *p* = 0.005) showed significant associations with intraoperative PCI score. Lower TBW/FFM was an independent predictor of major postoperative complications (OR 0.047, 95% CI 0.003–0.749, *p* = 0.031) and re-admission (OR 0.094, 95% CI 0.014–0.657, *p* = 0.017) within 30 days after surgery. Higher fat mass was also independently associated with a higher risk of major postoperative complications (OR 1.120, 95% CI 1.006–1.248, *p* = 0.039) and re-admission (OR 1.123, 95% CI 1.024–1.230, *p* = 0.013). Intraoperative PCI score > 20 (OR 4.489, 95% CI 1.191–16.917, *p* = 0.027) and re-admission within 30 days after surgery (OR 5.269, 95% CI 1.288–21.547, *p* = 0.021) independently predicted postoperative 1-year mortality.

**Conclusions:**

We demonstrate that preoperative BIA-derived TBW/FFM and fat mass were significantly correlated with metastatic extent, assessed by PCI score, in patients with peritoneal carcinomatosis. In addition, BIA-derived TBW/FFM and fat mass showed independent predictability for postoperative 30-day major complications and re-admission in patients undergoing CRS and HIPEC. Our findings suggest that assessment of BIA may improve discrete risk stratification in patients who are planned to receive CRS and HIPEC.

**Supplementary Information:**

The online version contains supplementary material available at 10.1186/s12957-023-03233-0.

## Background

With encouraging improvement in survival rates, hyperthermic intraperitoneal chemotherapy (HIPEC) added to cytoreductive surgery (CRS) has been emerging as a preferred treatment option and the last resort in patients diagnosed with peritoneal carcinomatosis. However, the procedure is highly associated with life-threatening complications that lead to perioperative death and an exponential increase in the healthcare burden [1]. A previous study reported a treatment-related mortality rate of 4.8% and postoperative morbidity of 21.5% after CRS and HIPEC [2]. Thus, risk stratification in terms of postoperative prognosis is the most important factor for success in this surgery. Although several scales including the peritoneal cancer index (PCI) score have been used [3], there is still a lack of comprehensive prognostic indicators that consider survival benefit and risk of postoperative morbidity.

Bioelectric impedance analysis (BIA), a commonly used technique for estimating body composition, is easy to use, reproducible, and non-invasive. Its utility as a potential marker of hydration and nutritional status and as a prognostic factor for clinical outcomes in diseased patients is being widely recognized [4]. The measure of body water distribution has been used to monitor volume status and prevent over-hydration in renal diseases and major surgeries [5, 6]. As a nutritional index, the BIA-derived phase angle (PhA) has shown predictability for mortality in renal failure patients [7]. The clinical significance of BIA-derived parameters has also been investigated in various types of cancer patients [8–12]. The PhA derived by BIA was reported to be associated with survival time in patients with non-small cell lung cancer [13], breast cancer [14], and colorectal cancer [15]. In addition, the BIA-derived fat-free mass (FFM) was associated with a prolonged hospital stay in colorectal cancer patients [16], and BIA-derived skeletal muscle mass was significantly related to the higher risk of respiratory complications in esophageal cancer patients [17]. However, there have been fewer attempts to interpret other meaningful parameters obtained from BIA in relation to tumor burden in advanced cancer patients. Further, the feasibility of BIA in estimating risk and benefit in major surgeries accompanying serious complications needs to be evaluated.

Therefore, we aimed to investigate whether BIA could provide prognostic information to improve the identification of patients who might potentially develop life-threatening complications after CRS and HIPEC. Since the PCI score is hitherto the most established prognostic indicator in this surgery, we explored the relationship between the BIA-derived parameters and the PCI score. Then, we investigated the predictability of selective perioperative data including BIA-derived parameters for postoperative major complications and readmission within 30 days after surgery as well as 1-year mortality.

## Methods

### Study population

**Fig. 1:**
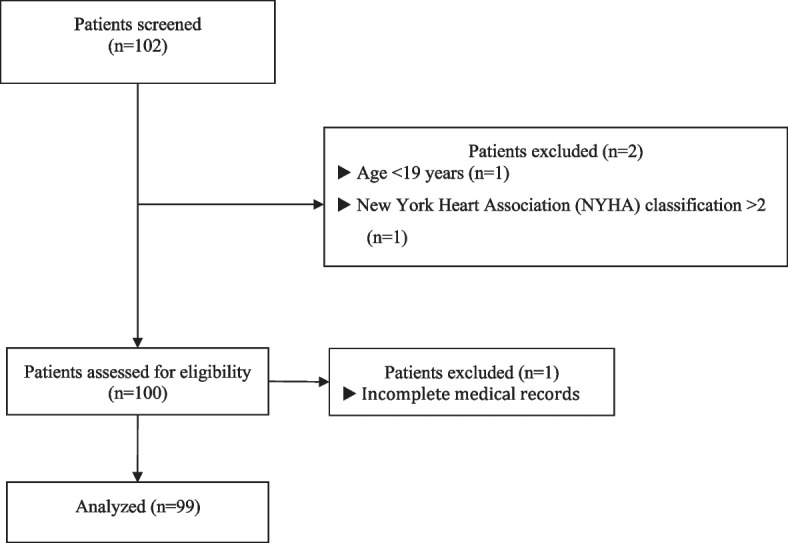
Flowchart of study enrollment

We retrospectively reviewed the electronic medical records of patients who received CRS and HIPEC at Gangnam Severance Hospital between March 2017 and August 2018 and enrolled patients with preoperative BIA test results (*n* = 102) (Fig. 1). The exclusion criteria were as follows: gynecological cancer, age < 19 years, conditions that may interfere with electrical property of tissues including end-stage renal disease, New York Heart Association classification of heart failure > 2, presence of infectious disease, and incomplete medical records. The study was approved by the Institutional Review Board of Gangnam Severance Hospital of Yonsei University Health System, Seoul, Korea (IRB protocol No. 3–2021-0435) and conducted according to the Declaration of Helsinki. The requirement for informed consent was waived due to the retrospective nature of this study.

### Surgical procedure

A standardized surgical technique was used in all cases [18]. CRS was conducted by resection of the metastatic organs from the primary cancer with a peritonectomy according to the Sugarbaker technique. Concurrent liver surgery including liver resection and/or radiofrequency ablation was performed. After CRS, HIPEC was subsequently performed using 35 mg/m^2^ of mitomycin-C mixed in 3 L of hypertonic solution (Dianeal, 1.5% dextrose peritoneal dialysis solution; Boxter Healthcare Corp., Deerfield, IL). Mitomycin-C was initially administered at 17.5 mg/m^2^ and 8.8 mg/m^2^ at 30 and 60 min, respectively. The mixed solution was circulated at 800–1000 mL/min by using a HIPEC pump (the Belmont Hyperthermia Pump; Belmont Medical Technologies, Billerica, MA, USA) to maintain a temperature of 42–43 °C for 90 min. Anastomosis of the resected bowel was performed after HIPEC.

### Perioperative data collection

All the demographic and perioperative clinical data were collected by a review of medical records. Baseline patient characteristics included age, sex, pre-existing comorbidities such as hypertension (HTN) and diabetes, preoperative European Cooperative Oncology Group Performance Status (ECOG PS), primary tumor origin, and preoperative PCI score assessed by computed tomography scan. Perioperative data included preoperative blood levels of albumin and creatinine, duration of surgery, intraoperative input and output, completeness of cytoreduction (CC) score, and intraoperative PCI score assessed by a surgeon intraoperatively. The PCI score is a sum of the points in 13 abdomino-pelvic regions [19]. Each region was graded as follows: 0 points, absence of tumor; 1 point, tumor < 5 mm in diameter; 2 points, tumor from 5 mm to 5 cm; and 3 points, tumor > 5 cm. The total score ranges from 0 to 39. CC0 or CC1 was considered to indicate complete cytoreduction, whereas incomplete cytoreduction was defined as CC2 or CC3.

Postoperatively, the length of intensive care unit (ICU) and hospital stay after surgery and postoperative 30-day morbidity including major postoperative complications, defined as Clavien–Dindo classification grades 3 and 4 that indicate complications requiring surgical, endoscopic, or radiological intervention and life-threatening complications requiring ICU management [20], re-admission, and mortality were recorded. The data on the mortality rate within 1 year of surgery and the overall mortality rate were also collected.

### BIA assessment

Measurement of body composition was performed using a portable BIA device with a 50-kHz alternating current, InBody S10 scanner (InBody Corp., Seoul, Republic of Korea), according to the manufacturer’s recommended instructions. All the BIA measurements were performed within 1 h before surgery with patients in a supine position. Touch-type electrodes were used: one on the thumb of each hand, one on each middle finger, and one on each inner ankle. For each patient, the data on the following parameters were obtained: total body water (TBW), intracellular water (ICW), extracellular water (ECW), ECW ratio (ECW/TBW), skeletal muscle mass, total body water/fat-free mass (TBW/FFM), and partitioned body components including protein, fat, and mineral, and PhA. All the measurements were performed by one well-trained anesthesiologist, and the measurement took around 2 min for each patient. The impedance values, displayed in real-time during BIA measurement, were closely monitored to ensure the accuracy of the measurement. If this impedance value did not meet the standard value range, it was considered a measurement error and was measured again. The BIA device was calibrated once a year according to the manufacturer’s instructions.

### Statistical analysis

All statistical analyses were performed using the SPSS version 23 (IBM Corp, Armonk, NY, USA) software. The normality of continuous variables was analyzed using the Kolmogorov–Smirnov test, and the variables were presented as mean. ± standard deviation (SD) or median (interquartile range [IQR]), as appropriate. Categorical variables were presented as the number of patients (percentage). Pearson’s correlation analysis was conducted to evaluate the relationship between the BIA-derived parameters and PCI scores. To select the parameters to be evaluated in predictive models, patients were divided into two groups depending on the presence or absence of postoperative morbidity. Continuous variables between each of the two groups were compared by independent *t*-tests for normally distributed variables; otherwise, the Mann–Whitney *U* test was used. Categorical variables were compared using the chi-squared or Fisher’s exact tests as appropriate.

The odds ratio (OR) and 95% confidence interval (CI) investigating the predictability of various perioperative factors and BIA-derived parameters for the occurrence of postoperative morbidities and mortality were assessed using logistic regression analysis. The following parameters were evaluated in the univariate analysis: age, sex, pre-existing hypertension and diabetes, ECOG PS, preoperative blood level of albumin, and BIA-derived parameters (TBW, ICW, ECW; protein, mineral, fat, skeletal muscle mass, TBW/FFM, PhA). The parameters with *p* < 0.2 between the groups according to the occurrence of each endpoint (major postoperative complications, re-admission, and mortality) were assessed. The variables with *p* < 0.2 were entered into the multivariate analysis. A *p*-value < 0.05 was considered statistically significant.

## Results

Among the 102 patients screened, 99 patients met the inclusion criteria and were analyzed (Fig. 1). Patient characteristics and perioperative data are described in Table 1. Forty-five patients were women, and the mean age was 56 (43–61) years. The mean (± SD) value of the body mass index (BMI) was 21.70 ± 3.76 kg/m^2^, and 22 patients (22.2%) had pre-existing HTN. Colorectal cancer accounted for 66.7% of the primary cancer type. The mean preoperative serum albumin level was 3.98 ± 0.51 g/dL, and BIA-derived TBW was 32.6 (27.1–38.6) L. The amounts of protein, mineral, and fat were 8.81 ± 1.86 kg, 3.08 ± 0.62 kg, and 16.21 ± 7.31 kg, respectively, in weight. The mean skeletal muscle mass was 24.56 ± 5.59 kg, and the median TBW/FFM was 73.7 (73.4–73.9) %. The mean PhA was 5.22 ± 0.95°. The median intraoperative PCI score was 14 (6.5–28.5), and the proportion of patients with intraoperative PCI score > 20 was 36.4%. The median CC score was 0 (0–2), and incomplete cytoreduction defined as CC score 2–3 was observed in 27.3% of patients. The rate of patients with major postoperative complications was 11.1%, and the re-admission rate was 16.2%. The 1-year mortality rate was 13.1%, and the overall survival rate was 31.3%. The median follow-up duration was 1097 (688–1293) days.

**Table 1:** Baseline characteristics and perioperative data

Variables	Total (*N* = 99)
Age (years)	56 (43–61)
Female sex	45 (45.5%)
Body mass index (kg/m^2^)	21.70 ± 3.76
Pre-existing co-morbidity	
Hypertension	22 (22.2%)
Diabetes mellitus	9 (9.1%)
Cerebrovascular accident	2 (2.0%)
Chronic kidney disease	1 (1.0%)
European Cooperative Oncology Group Performance Status	1 (0–2)
Primary tumor type	
Colorectal cancer	66 (66.7%)
Mesothelioma or pseudomyxoma peritonei	8 (8.1%)
Stomach cancer	18 (18.2%)
Appendiceal cancer	3 (3.0%)
Others	4 (4.0%)
Preoperative albumin (g/dL)	3.98 ± 0.51
Bioelectric impedance analysis parameters	
Total body water (L)	32.6 (27.1–38.6)
Intracellular water (L)	20.36 ± 4.30
Extracellular water (L)	13.0 (10.8–14.8)
Protein (kg)	8.81 ± 1.86
Mineral (kg)	3.08 ± 0.62
Fat (kg)	16.21 ± 7.31
Skeletal muscle mass (kg)	24.56 ± 5.59
Total body water/fat-free mass (%)	73.7 (73.4–73.9)
Phase angle (°)	5.22 ± 0.95
Preoperative PCI score	12 (6–18)
Preoperative PCI score > 20	16 (16.2%)
Preoperative PCI score 15–20	22 (22.2%)
Preoperative PCI score < 15	61 (61.6%)
Intraoperative PCI score	14 (6.5–28.5)
Intraoperative PCI score > 20	36 (36.4%)
Intraoperative PCI score 15–20	11 (11.1%)
Intraoperative PCI score < 15	52 (52.5%)
Completeness of cytoreduction score	0 (0–2)
Completeness of cytoreduction score 0–1	72 (72.7%)
Completeness of cytoreduction score 2–3	27 (27.3%)
Surgery time (min)	546.43 ± 21.95
Intraoperatively administered fluid (mL)	6350 (4500–8300)
Intraoperatively transfused packed red blood cells (mL)	0 (0–500)
Intraoperative urine output (mL)	1090 (685–1600)
Intraoperative bleeding (mL)	900 (400–1800)
Length of intensive care unit stay (day)	1 (1–1)
Length of hospital stay (days)	15 (13–18)
Postoperative 30-day morbidity	
Clavien–Dindo grades 3 and 4	11 (11.1%)
Re-admission	16 (16.2%)
In-hospital mortality	2 (2.0%)
One-year mortality	13 (13.1%)
Overall mortality	31 (31.3%)

Values are presented as mean ± standard deviation, median (interquartile range), or number of patients (%)

*PCI score*, peritoneal cancer index score

The correlation between the BIA parameters and intraoperative PCI score analyzed by Pearson’s correlation analysis is shown in Table 2. The amount of minerals (*r* = 0.224, *p* = 0.027), fat (*r* =  − 0.202, *p* = 0.048), and TBW/FFM (*r* =  − 0.280, *p* = 0.005) showed significant associations with intraoperative PCI score. The analysis of the correlation between the BIA parameters and preoperative PCI score also showed similar results (Additional file 1: Table S1). In subgroup analysis for colorectal cancer patients, BIA-derived mineral and TBW/FFM showed significant association with intraoperative PCI scores (Additional file 2: Table S2).

**Table 2:** Association between BIA parameters and intraoperative peritoneal cancer index score

BIA parameters	Intraoperative PCI score	
	*r*	*p*-value
Total body water	0.089	0.386
Intracellular water	0.073	0.475
Extracellular water	0.115	0.260
Protein	0.073	0.477
Mineral	0.224	0.027
Fat	− 0.202	0.048
Muscle	0.073	0.478
Total body water/fat-free mass	− 0.280	0.005
Phase angle	− 0.059	0.564

*BIA* bioelectric impedance analysis, *PCI* peritoneal cancer index, *r* correlation coefficient obtained from Pearson’s correlation analysis

Table 3 describes the logistic regression models to predict postoperative 30-day morbidity. In the univariate analysis of predictors for major postoperative complications, HTN, ECOG PS, BIA-derived mineral, fat, TBW/FFM, and PhA had a *p*-value < 0.2. In the multivariate analysis of these variables, higher body fat (OR 1.120, 95% CI 1.006–1.248, *p* = 0.039) and lower TBW/FFM (OR 0.047, 95% CI 0.003–0.749, *p* = 0.031) were independent predictors for the occurrence of major postoperative complications. In the univariate analysis of predictors for re-admission, HTN and BIA-derived fat mass and TBW/FFM had a *p*-value < 0.2. In the multivariate analysis of these variables, higher body fat (OR 1.123, 95% CI 1.024–1.230, *p* = 0.013) and lower TBW/FFM (OR 0.094, 95% CI 0.014–0.657, *p* = 0.017) as well as HTN remained independent predictors.

**Table 3:** Predictive power of selective variables including BIA parameters for postoperative 30-day morbidity

	Major postoperative complications (*N* = 11/99)				Re-admission (*N* = 16/99)			
	Univariate		Multivariate		Univariate		Multivariate	
	OR (95% CI)	*p*-value	OR (95% CI)	*p*-value	OR (95% CI)	*p*-value	OR (95% CI)	*p*-value
Age	1.023 (0.969–1.081)	0.413			0.995 (0.953–1.039)	0.814		
Body mass index	0.989 (0.836–1.169)	0.892			1.015 (0.880–1.171)	0.838		
Hypertension	3.480 (0.949–12.764)	0.060	4.080 (0.907–18.341)	0.067	4.929 (1.583–15.349)	0.006	5.772 (1.594–20.903)	0.008
Diabetes mellitus	1.000 (0.113–8.849)	1.000			1.551 (0.291–8.253)	0.607		
ECOG PS	1.644 (0.810–3.336)	0.169	1.761 (0.807–3.840)	0.155	1.287 (0.702–2.362)	0.415		
Preoperative albumin	0.584 (0.175–1.950)	0.382			1.299 (0.444–3.801)	0.633		
BIA parameters								
TBW	1.043 (0.949–1.146)	0.379			1.021 (0.943–1.107)	0.606		
ICW	1.071 (0.925–1.240)	0.359			1.034 (0.912–1.171)	0.603		
ECW	1.112 (0.857–1.442)	0.423			1.058 (0.848–1.320)	0.617		
Protein	1.179 (0.840–1.657)	0.342			1.079 (0.809–1.440)	0.605		
Mineral	2.011 (0.761–5.315)	0.159	0.637 (0.147–2.758)	0.547	0.596 (0.685–3.719)	0.279		
Fat	1.065 (0.981–1.157)	0.134	1.120 (1.006–1.248)	0.039	1.099 (1.020–1.185)	0.013	1.123 (1.024–1.230)	0.013
Skeletal muscle mass	1.055 (0.942–1.180)	0.356			1.026 (0.932–1.129)	0.603		
TBW/FFM	0.168 (0.028–1.001)	0.050	0.047 (0.003–0.749)	0.031	0.339 (0.073–1.579)	0.168	0.094 (0.014–0.657)	0.017
PhA	1.903 (0.892–4.058)	0.096	1.718 (0.611–4.833)	0.305	1.200 (0.671–2.147)	0.538		
Intraoperative PCI > 20	1.478 (0.417–5.243)	0.545			1.893 (0.642–5.583)	0.248		

Values are presented as odds ratios (95% confidence intervals)

*OR* odds ratio, *CI* confidence interval, *ECOG PS* European Cooperative Oncology Group Performance Status, *BIA* bioelectric impedance analysis, *TBW* total body water, *ICW* intracellular water, *ECW* extracellular water, *TBW/FFM* total body water/fat-free mass, *PhA* phase angle, *PCI* peritoneal cancer index

Table 4 describes the logistic regression analysis to predict 1-year mortality after surgery. In the univariate analysis of predictors, intraoperative PCI scores > 20, 30-day major postoperative complications, and 30-day re-admission had a *p*-value < 0.2. In the multivariate analysis of these variables, intraoperative PCI score > 20 (OR 4.489, 95% CI 1.191–16.917, *p* = 0.027) and re-admission (OR 5.269, 95% CI 1.288–21.547, *p* = 0.021) remained independent predictors.

**Table 4:** Predictive power of selective variables including BIA parameters for 1-year mortality

	1-year mortality (*N* = 13/99)			
	Univariate		Multivariate	
	OR (95% CI)	*p*-value	OR (95% CI)	*p*-value
Age	1.002 (0.955–1.051)	0.937		
Hypertension	1.679 (0.463–6.083)	0.430		
Diabetes mellitus	0.813 (0.093–7.087)	0.851		
ECOG PS	0.896 (0.449–1.789)	0.756		
Preoperative albumin	0.946 (0.302–2.964)	0.924		
BIA parameters				
Protein	1.162 (0.847–1.594)	0.351		
Fat	0.973 (0.895–1.058)	0.526		
Mineral	1.683 (0.676–4.194)	0.263		
Skeletal muscle mass	1.052 (0.947–1.168)	0.342		
TBW/FFM	0.720 (0.133–3.898)	0.703		
PhA	1.071 (0.573–1.999)	0.831		
Intraoperative PCI > 20	4.750 (1.342–16.807)	0.016	4.489 (1.191–16.917)	0.027
30-day major postoperative complications	2.925 (0.665–12.865)	0.156	1.469 (0.269–8.007)	0.657
30-day postoperative re-admission	6.514 (1.822–23.295)	0.004	5.269 (1.288–21.547)	0.021

Values are presented as odds ratios (95% confidence intervals)

*OR* odds ratio, *CI* confidence interval, *ECOG PS* European Cooperative Oncology Group Performance Status, *BIA* bioelectric impedance analysis, *TBW/FFM* total body water/fat-free mass, *PhA*, phase angle; *PCI*, peritoneal cancer index

## Discussion

Body composition analysis is emerging as a reliable prognostic indicator in various cancer cohorts [9, 12]. However, its utility has never been explored in patients with advanced cancer undergoing surgical treatment. In the current study on 99 patients undergoing CRS and HIPEC surgery in our institution for 2 years, we found that BIA-derived body fat mass and TBW/FFM were closely associated with the metastatic extent and could discriminate patients at high risk for serious postoperative morbidity. This is the first study to report significant implications of BIA-derived body composition in patients receiving CRS and HIPEC surgery for peritoneal carcinomatosis.

Body fat mass was negatively correlated with metastatic extent indicated by PCI score, which can be explained by the fat loss associated with cancer cachexia [21–24]. Cancer patients experience adipose atrophy via increased lipolysis, decreased lipid deposition and lipogenesis, and increased mitochondrial fatty acid oxidation as the disease progresses [23]. Pro-inflammatory cytokines produced by tumor and adipose tissue itself may also contribute to the depletion of adipose tissue [24]. Thus, disease severity might be reflected by the degree of fat loss in advanced cancer, as was seen in our result of the strong association between lower fat mass with PCI score. On the other hand, BIA-derived skeletal muscle mass was not associated with PCI score or any of the clinical endpoints in the current study. It is consistent with a recent report on BIA-assessed cachexia in cancer patients [25]. In that study, BIA-derived fat mass and fat mass index could better distinguish cancer stages (stage I vs. stages II–IV and stages I–II vs. stages III–IV) than the indices of skeletal muscle mass in both sexes. Moreover, despite the lack of solid consensus on whether fat- or muscle loss occurs first during cancer cachexia, there is considerable evidence supporting the more rapid occurrence of fat loss than lean tissue loss during disease progression, majority of which were assessed by BIA [26–28]. Several experimental studies also demonstrated that fat loss occurred prior to muscle loss in cancer [29, 30]. Our findings may support the clinical role of fat loss as a nutritional indicator encompassing perioperative prognosis in peritoneal carcinomatosis patients undergoing major surgery. On the other hand, higher fat mass was observed to be an independent predictor for postoperative major complications and re-admission in our study. This is consistent with the findings of previous studies that identified large amounts of fat and obesity as risk factors for more surgical complications, re-admission, and re-surgery after abdominal cancer surgery [31, 32]. Technical difficulties during surgery along with metabolic and immunological factors associated with fat above the standard amount may cause such problems [31, 32]. Since the current cohort undergoing CRS and HIPEC included patients who were relatively newly diagnosed, obese and pre-obese patients were present despite the high cancer stage. Nevertheless, this finding should be interpreted with caution because interactions between fat and cancer may differ according to the primary cancer origin. Further investigations in a large population are needed to determine the target values for optimal nutritional management.

The value of TBW/FFM has long been recognized to be increased in nearly all disease states, apart from few acute conditions, as a result of expansion of ECW and contraction of the body cell mass [33]. However, its pattern and clinical implication in cancer patients are not completely known. In the current study, lower TBW/FFM was associated with higher PCI score. Considering that the amount of bone mineral, skeletal muscle mass, and soft lean mass that constitute FFM were not correlated with the PCI score, we could assume that tumor burden could have resulted in a difference in FFM in the current results. Indeed, FFM is characterized by high electrical conductivity and low impedance [4, 34]. A recent ex vivo mouse study revealed that cancerous tissues had lower impedance than normal tissues, which were assessed by electrical impedance spectroscopy [35]. Moreover, the majority of malignant solid tumors in humans showed higher electrical conductivity and lower impedance compared with normal tissues [36, 37]. In cases where large masses of tumor spread to the abdominal organs as well as the omentum and peritoneum, the tumor may possibly affect the FFM value that is assessed by electrical impedance analysis. Since the value of TBW/FFM is relatively stable and exists in a narrow range, even a small change may reflect meaningful alterations in homeostasis [38]. Significant predictability of low TBW/FFM for the occurrence of major postoperative complications and re-admission would be understood in this context, since a higher tumor burden would lead to aggressive debulking procedures, which would definitely raise the risk of complications that require intervention as well as re-admission. The ORs of 0.047 and 0.094 in the logistic regression analysis suggest that a small difference in TBW/FFM may discern the risk of postoperative complications and re-admission after surgery as well. Despite such a potential predictability for perioperative outcomes, lower TBW/FFM was not associated with 1-year mortality. However, re-admission was an independent predictor of 1-year mortality, which is consistent with a recent report on 342 patients receiving CRS and HIPEC [39]. Major postoperative complication was also associated with shorter survival after CRS and HIPEC in 113 patients with colorectal or appendicular carcinomatosis [40]. Moreover, intraoperative PCI score > 20 predicted 1-year mortality in the current study. Therefore, we could assume that lower TBW/FFM assessed by BIA could be helpful, at least indirectly, in predicting survival after surgery.

BIA is a non-invasive, easy-to-use, and cost-effective tool that can be used in cancer patients [10–12]. However, BIA is known to be influenced by several confounding factors [41], such as electrode placement and type [42, 43], patient posture [44], or food intake [45]. Therefore, BIA measurement should be conducted carefully, and the results should be interpreted with caution. In this study, we measured BIA under the same conditions, preoperative fasting state, and supine position, with close monitoring of the impedance values during measurement, which made our data reliable. Numerous studies have validated that BIA measurement shows high reliability and reproducibility when properly measured [4, 46–48]. In addition, the BIA-derived parameters have been reported to be closely associated with prognosis in cancer patients [13–17]. BIA may be used as an auxiliary tool for risk stratification in cancer patients in conjunction with other assessments.

There are several limitations to the current study. First, BIA is based on the numbers of assumptions; thus, biological variation in patients may change these assumptions and reduce its accuracy. The measurement of FFM also partly depends on the value of TBW assuming a constant ratio between them, while it is calculated with the use of additional anthropometric equations, which result in individual values for each patient. Therefore, caution is needed while interpreting our results regarding TBW/FFM. Second, we used the results of a single BIA measurement. Since BIA is affected by various confounding factors, it would have been more accurate to measure it multiple times. Third, our data did not include postoperative BIA measurements. Future studies are warranted to analyze the changes in BIA parameters before and after surgery, to demonstrate the relationship between BIA parameters and tumor burden. Fourth, the effect of neoadjuvant treatment has not been analyzed. Since neoadjuvant treatment may have affected BIA parameters, this may have been a confounding factor. Future studies are warranted to conduct a more accurate analysis considering the effect of neoadjuvant treatment. Fifth, our study was conducted at a single institution and analyzed a relatively small sample size with heterogeneous primary tumor origins. Our preliminary results require validation in further studies with larger populations and various types of cancers.

## Conclusion

BIA-derived body fat mass and TBW/FFM were correlated with the extent of peritoneal metastasis and showed independent predictability for postoperative 30-day morbidity after CRS and HIPEC. Measuring the BIA parameters may potentially help to predict the tumor burden and screen high-risk patients in advanced cancer patients with peritoneal carcinomatosis. However, the clinical significance of BIA parameters in advanced cancer patients should be verified in further studies with larger populations and various types of cancers.

### Supplementary Information

**Additional file 1: Table S1. **Association between BIA parameters and preoperative peritoneal cancer index score. BIA, bioelectric impedance analysis; PCI, peritoneal cancer index; r, correlation coefficient obtained from Pearson’s correlation analysis.

**Additional file 2: Table S2. **Subgroup analysis for association between BIA parameters and intraoperative peritoneal cancer index score. BIA, bioelectric impedance analysis; PCI, peritoneal cancer index; r, correlation coefficient obtained from Pearson’s correlation analysis.

## Data Availability

The datasets used and/or analyzed during the current study are available from the corresponding author upon reasonable request.

## Abbreviations

HIPEC: Hyperthermic intraperitoneal chemotherapy
CRS: Cytoreductive surgery
PCI: Peritoneal cancer index
BIA: Bioelectric impedance analysis
HTN: Hypertension
ECOG PS: European Cooperative Oncology Group Performance Status
CC: Completeness of cytoreduction
ICU: Intensive care unit
TBW: Total body water
ICW: Intracellular water
ECW: Extracellular water
TBW/FFM: Total body water/fat-free mass
PhA: Phase angle
OR: Odds ratio
CI: Confidence interval
BMI: Body mass index
