# Supplementary material for: Prognostic role of body composition in peritoneal carcinomatosis patients undergoing cytoreduction and hyperthermic intraperitoneal chemotherapy
Source: World J Surg Oncol. 2023 Oct 27;21:345. doi: 10.1186/s12957-023-03233-0 (PMC10604686; doi:10.1186/s12957-023-03233-0)
Supplement: Supplementary file 1 — Additional file 1: Table S1. Association between BIA parameters and preoperative peritoneal cancer index score. BIA, bioelectric impedance analysis; PCI, peritoneal cancer index; r, correlation coefficient obtained from Pearson’s correlation analysis. [file 12957_2023_3233_MOESM1_ESM.docx]

Supplementary Table S1. Association between BIA parameters and preoperative peritoneal cancer index score

| **BIA parameters** | Preoperative PCI score | |
| --- | --- | --- |
|  | r | *p*-value |
| Total body water | 0.112 | 0.281 |
| Intracellular water | 0.102 | 0.326 |
| Extracellular water | 0.128 | 0.218 |
| Protein | 0.100 | 0.337 |
| Mineral | 0.244 | 0.017 |
| Fat | -0.274 | 0.007 |
| Muscle | 0.101 | 0.330 |
| Total body water/Fat free mass | -0.300 | 0.003 |
| Phase angle | 0.037 | 0.725 |

BIA, bioelectric impedance analysis; PCI, peritoneal cancer index; r, correlation coefficient obtained from Pearson’s correlation analysis.
