# Supplementary material for: Prognostic role of body composition in peritoneal carcinomatosis patients undergoing cytoreduction and hyperthermic intraperitoneal chemotherapy
Source: World J Surg Oncol. 2023 Oct 27;21:345. doi: 10.1186/s12957-023-03233-0 (PMC10604686; doi:10.1186/s12957-023-03233-0)
Supplement: Supplementary file 2 — Additional file 2: Table S2. Subgroup analysis for association between BIA parameters and intraoperative peritoneal cancer index score. BIA, bioelectric impedance analysis; PCI, peritoneal cancer index; r, correlation coefficient obtained from Pearson’s correlation analysis. [file 12957_2023_3233_MOESM2_ESM.docx]

Supplementary Table S2. Subgroup analysis for association between BIA parameters and intraoperative peritoneal cancer index score

| **BIA parameters** | **Colorectal cancer (N = 66)** | | **Stomach cancer (N = 18)** | |
| --- | --- | --- | --- | --- |
|  | Intraoperative PCI score | | Intraoperative PCI score | |
|  | r | *p*-value | r | *p*-value |
| Total body water | 0.169 | 0.177 | -0.192 | 0.444 |
| Intracellular water | 0.152 | 0.228 | -0.191 | 0.448 |
| Extracellular water | 0.199 | 0.113 | -0.192 | 0.445 |
| Protein | 0.149 | 0.235 | -0.182 | 0.469 |
| Mineral | 0.300 | 0.015 | -0.087 | 0.730 |
| Fat | -0.145 | 0.251 | -0.417 | 0.085 |
| Muscle | 0.151 | 0.230 | -0.191 | 0.449 |
| Total body water/Fat free mass | -0.313 | 0.011 | -0.301 | 0.225 |
| Phase angle | -0.022 | 0.860 | -0.142 | 0.573 |

BIA, bioelectric impedance analysis; PCI, peritoneal cancer index; r, correlation coefficient obtained from Pearson’s correlation analysis.
